# The status of RNAi-based transgenic research in plant nematology

**DOI:** 10.3389/fmicb.2014.00760

**Published:** 2015-01-12

**Authors:** Tushar K. Dutta, Prakash Banakar, Uma Rao

**Affiliations:** Division of Nematology, Indian Agricultural Research InstituteNew Delhi, India

**Keywords:** gene silencing, dsRNA, siRNA, root-knot nematodes, cyst nematodes, host-delivered RNAi

## Abstract

With the understanding of nematode-plant interactions at the molecular level, new avenues for engineering resistance have opened up, with RNA interference being one of them. Induction of RNAi by delivering double-stranded RNA (dsRNA) has been very successful in the model non-parasitic nematode, *Caenorhabditis elegans*, while in plant nematodes, dsRNA delivery has been accomplished by soaking nematodes with dsRNA solution mixed with synthetic neurostimulants. The success of *in vitro* RNAi of target genes has inspired the use of *in planta* delivery of dsRNA to feeding nematodes. The most convincing success of host-delivered RNAi has been achieved against root-knot nematodes. Plant-mediated RNAi has been shown to lead to the specific down-regulation of target genes in invading nematodes, which had a profound effect on nematode development. RNAi-based transgenics are advantageous as they do not produce any functional foreign proteins and target organisms in a sequence-specific manner. Although the development of RNAi-based transgenics against plant nematodes is still in the preliminary stage, they offer novel management strategy for the future.

## Introduction

To date, more than 4100 species of plant-parasitic nematode (PPN) have been reported (Jones et al., 2013) and collectively they are one of the major limiting factors in crop production worldwide besides insects and pathogens. Estimated damage by PPNs may be up to $US173 billion every year (Elling, 2013). PPNs are microscopic soil-borne animals; all species may not cause appreciable crop loss or symptom development as other pests and pathogens do. Sedentary endoparasites like root-knot and cyst nematodes can potentially cause damage to crops. Being obligate biotrophic pathogens, they establish an intimate relationship with their host plant. Secretions from their esophageal glands contain proteins that induce specialized feeding cells (giant cell and syncytia) in the host root, which serves as the nutrient sink for nematode development and reproduction (Davis et al., 2004). Infected plants slowly suffer damage due to an interruption in the translocation of water and nutrients from the root to the shoot. In addition, considerable damage to crop plants is caused by sedentary semi-endoparasite such as *Rotylenchulus reniformis*; migratory endoparasites such as *Pratylenchus* spp., *Radopholus* spp., *Bursaphelenchus xylophilus*; above ground parasite such as *Aphelenchoides besseyi* etc. (Jones et al., 2013). Therefore, to combat these pests, adoption of novel environmentally-friendly and cost-effective management strategies is necessary.

The discovery of RNA interference (RNAi) in the free-living nematode, *Caenorhabditis elegans*, in which double-stranded RNA (dsRNA) triggers the post-transcriptional silencing of endogenous genes with homologous sequences, has provided a revolutionary tool to analyze gene function (Fire et al., 1998). In *C. elegans*, exogenous dsRNA can be introduced into the nematode digestive system by feeding on dsRNA-expressing bacterium, *Escherichia coli*. This dsRNA then spreads systemically to neighboring tissues through the action of the SID-2 and SID-1 transmembrane proteins, followed by the cleavage of dsRNA by the RNaseIII enzyme, Dicer, into 21–25 nucleotide long short interfering RNAs (siRNAs) in the cell cytoplasm. Attachment of siRNAs to the RNA-induced silencing complex (RISC) separates the strands of siRNAs and incorporates the siRNAs into the active RISC. These siRNAs then target mRNA molecule in a sequence-specific manner and the RISC cleaves them, inducing a gene silencing effect (Rosso et al., 2009). Similar mechanisms of action appear to function in PPNs as well. Therefore, RNAi can be utilized as a powerful tool to develop RNAi-based transgenic plants to combat the menace of PPNs.

## *in vitro* RNAi

Initially, dsRNAs of the target gene were introduced into the free-living worm, *C. elegans*, by means of (i) microinjection, (ii) feeding with transformed *E. coli* cells expressing target gene dsRNA and (iii) soaking of worms in dsRNA solution, to assess the gene silencing effect in distal tissues. However, in the case of PPNs using similar methods has been a major challenge. Introduction of dsRNA to PPNs using microinjection could not be achieved consistently. Furthermore, PPN's obligatory parasitic nature limits the options for *in vitro* delivery. Second stage juveniles and eggs, the only free-living stages of the sedentary species of PPN, have been used for *in vitro* RNAi in most of the cases using a soaking method of delivery (Lilley et al., 2012).

**Figure 1:**
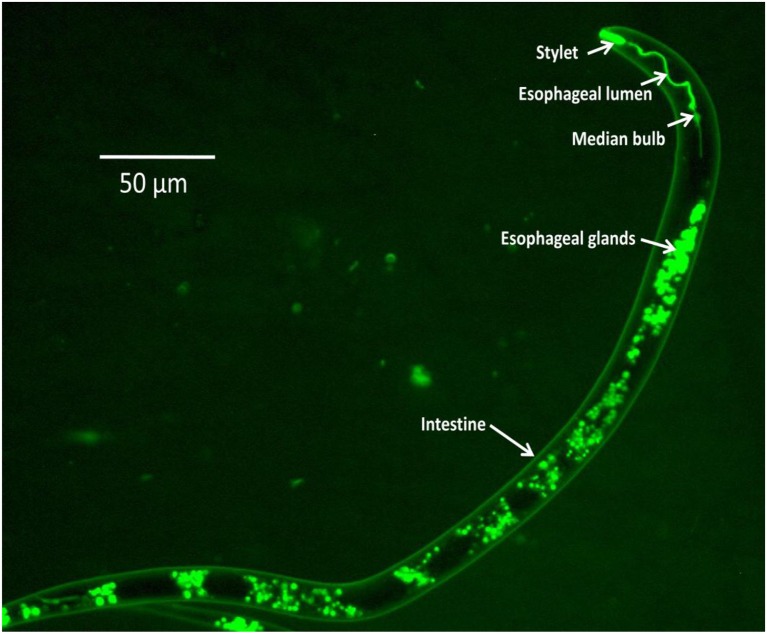
**FITC uptake in different body parts of *M. graminicola* J_2_16h after treatment with the neurotransmitter serotonin (50 mM) in soaking buffer**.

The availability of genome information and transcriptomic data of several economically important nematodes (Martin et al., 2012) has led to the identification of candidate genes that have been targeted for RNAi experiments. Silencing of target genes through *in vitro* RNAi in PPNs was first achieved in infective juveniles of the cyst nematodes, *Globodera pallida* and *Heterodera glycines*, through neurostimulant-mediated oral ingestion of dsRNA molecules (Urwin et al., 2002). Monitoring of dsRNA uptake in the nematode body is achieved by the addition of a fluorescent chemical, FITC (fluorescein isothiocyanate) in the soaking solution or by using fluorescently-labeled dsRNAs (Figure 1). Using *in vitro* RNAi strategies has demonstrated down-regulation of targeted genes with corresponding phenotypic changes such as decline in parasitic success, reduction in fecundity, motility inhibition, reduced host location ability, and decline in penetration and reproduction in host roots. These results have been demonstrated successfully in root-knot, cyst, lesion, pine wilt, burrowing, and white tip nematodes (Lilley et al., 2012; Cheng et al., 2013; Tan et al., 2013). Functional validation of several nematode genes through *in vitro* RNAi has paved the way for the application of host-induced gene silencing (HIGS) strategies targeting various PPNs in different crop plants.

## *in planta* RNAi

For host-delivered RNAi, plants are genetically modified to express dsRNA molecules with sequence derived from the target gene. RNAi constructs are made by cloning a fragment of the target gene coding sequence in sense and antisense orientation, separated by an intron/spacer region, under the control of a constitutive or tissue-specific promoter. Upon transcription, sense and antisense strands of the target gene complement each other and form an intron spliced hairpin RNA of which a significant portion is dsRNA (Helliwell and Waterhouse, 2003). Successful *in vitro* experiments have shown that PPNs can ingest dsRNA molecules on its own. Subsequently, dsRNA molecules can also be processed by the plant Dicer enzymes into siRNA molecules (Bakhetia et al., 2005). As the nematode feeds on the plant during its parasitic phase, it consequently assures the introduction of dsRNA and/or siRNA molecules into the nematode's digestive system. Indeed, transfer of dsRNA and/or siRNAs from plants to various pathogens has been demonstrated in a number of experimental systems and is often referred to as host-induced gene silencing (Koch and Kogel, 2014).

The first successful demonstration of host-delivered RNAi was accomplished by targeting integrase and splicing factor genes of *M. incognita*. Tobacco transgenic plants constitutively expressing dsRNA of these genes resulted in a reduction of nematode establishment due to the repression of the targeted nematode mRNA (Yadav et al., 2006). In another report, transgenic soybean expressing *PRP17* dsRNA caused 53% and 79% reduction in infection and reproduction of *H. glycines*, respectively. RNAi of the *Cpn1* gene (a homolog of a *C. elegans* embryonic lethal gene), displayed up to 95% reduction in the number of egg masses of *H. glycines* infecting soybean. A similar strategy was deployed to silence the genes encoding a β-subunit of the coatomer (COPI) complex and a pre-mRNA splicing factor, resulting in the reduced infection and development of *H. glycines* on the transgenic soybean (Li et al., 2010a,b). As reported by Klink et al. (2009), *in planta* RNAi targeting four embryonic lethal genes of *C. elegans*—encoding ribosomal protein 3a and 4, a spliceosomal SR protein and synaptobrevin*—*expressed as tandem inverted repeats in transgenic soybean inhibited female development of *H. glycines*. More recently, tomato hairy roots expressing a hairpin of the *mj-far-1* (fatty acid and retinol binding protein) gene of *M. javanica* led to 80% reduction in transcript abundance in the feeding nematodes and a significant reduction in giant cell number due to impaired development of females (Iberkleid et al., 2013). Based on these findings, it may seem tempting to choose housekeeping genes for RNAi targeting, but some precautions should be taken before selecting such kind of targets as most of these genes are highly conserved across different species and could potentially have off-target effects on host plants or other beneficial organisms including humans.

Nematode specific genes pose the most attractive targets for nematode management through host-delivered RNAi, as the observed phenotypic effect can be directly correlated to the silencing specificity. When a parasitism gene, *16D10*, was used for RNAi in the model plant, *Arabidopsis thaliana*, a significant decline (63–90%) in gall number and a corresponding decline in fecundity of *M. incognita* was reported (Huang et al., 2006). The high level of identity between the *16D10* sequences of various *Meloidogyne* species, including *M. incognita, M. javanica, M. arenaria*, and *M. hapla*, suggests that *16D10* could be exploited as a candidate gene for developing RNAi-based transgenics against a broader range of nematode species. In the case of cyst nematodes, 68% reduction in fecundity was reported in transgenic soybean plants expressing dsRNA of the *MSP* (major sperm protein) gene of *H. glycines* (Steeves et al., 2006). Likewise, four parasitism genes of *H. schachtii* have been targeted to engineer Arabidopsis plants for host-delivered RNAi (Sindhu et al., 2009).

Soybean plants expressing an RNA hairpin targeting the tyrosine phosphatase gene of *M. incognita* supported 92% fewer galls than control plants (Ibrahim et al., 2011). When the *M. incognita* calreticulin gene, *Mi-CRT* (Jaouannet et al., 2013) and a parasitism gene, *8D05* (Xue et al., 2013), were targeted for RNAi in Arabidopsis plants, a significant reduction in gall number and developing females were observed. Silencing of two neuropeptide genes of *M. incognita, flp-14*, and *flp-18*, through host-induced RNAi in tobacco, reduced nematode infectivity and multiplication ability by 50–80%. As the transformed plants did not show any visible phenotypic variation, it was speculated that RNAi constructs of *flp*s did not have any off-target effects (Papolu et al., 2013).

In a recent report, three potato cultivars (cvs Désirée and Russet Burbank, and the advanced breeding line, PA99N82–4), expressing an RNAi construct targeting a putative effector gene, *Mc16D10L* (an ortholog of *M. incognita 16D10* gene), exhibited resistance to *M. chitwoodi*. Additionally, the 16D10i-2 RNAi transgene did not interfere with the natural resistance conferred by the *R*_*Mc1(blb)*_gene, which has been introgressed into PA99N82–4. As RNAi-induced resistance against *M. chitwoodi* reached comparable levels in all cultivars tested, genotype specific factors are unlikely to limit the use of RNAi in a broad range of germplasm (Dinh et al., 2014a). Furthermore, stable transgenic lines of *A. thaliana* and potato expressing dsRNA of *Mc16D10L*, have also been reported to confer resistance to *M. chitwoodi* (Dinh et al., 2014b). Nematodes feeding on these transgenics also appeared to transmit the downregulation of the targeted gene to their offspring and this effect was maintained over several generations (Dinh et al., 2014a,b). Therefore, as seen in *C. elegans* (Grishok et al., 2000), plant-mediated RNAi effects could become systemic and heritable in PPNs.

## Pros and cons of *in planta* RNAi

Based on the above reports, *in planta* dsRNA/siRNA uptake in PPNs seems to be effective due to specific down-regulation of target genes and nematodes infecting transgenic plants have shown reduced parasitic success with a consequent reduction in the number of mature females in different RNAi lines (Table 1). According to their functions, genes targeted for *in planta* RNAi can be broadly divided into three categories: parasitism-related genes, development-related genes and housekeeping genes. When the effector or parasitism genes such as *16D10, MiCRT, 8D05, HYP, 3B05, 4G06, 8H07*, and *10A06*, which are expressed in the sub-ventral or dorsal esophageal glands of different PPNs, were knocked down, notable interference in the parasitism process was reported (Huang et al., 2006; Sindhu et al., 2009; Jaouannet et al., 2013; Xue et al., 2013; Eves-van den Akker et al., 2014). Plant-mediated silencing of the genes necessary for nematode development and reproduction including *flp, MSP, Cpn-1, Y25, Fib1, Prp-17*, tyrosine phosphatase, mitochondrial stress-70 protein precursors, lactate dehydrogenase etc., also had deleterious effect on the nematode survival (Steeves et al., 2006; Li et al., 2010a,b; Ibrahim et al., 2011; Papolu et al., 2013). Among the housekeeping genes targeted, host-delivered RNAi targeting of lethal genes such as integrase, splicing factor, ribosomal protein-3a, 4, spliceosomal SR protein, coatomers, *Pv010, Mi-Rpn7* etc. affected the reproductive fitness and parasitic success of the invading nematodes (Yadav et al., 2006; Klink et al., 2009; Niu et al., 2012; Walawage et al., 2013). Tobacco plants expressing RNAi construct of *MjTIS-11* (*M. javanica* putative transcription factor) did not cause any phenotypic effect in the invading nematodes, although transcript suppression and siRNA generation were detected in transgenic plants (Fairbairn et al., 2007). However, this may not be surprising as not all targeted genes will necessarily lead to phenotypes due to genetic redundancy.

**Table 1:** **Summary of *in planta* RNAi against PPN genes and the resulting phenotypes**.

**Target gene**	**Species**	**Host plant**	**Observed phenotype**	Time point^*^	**References**
Integrase, Splicing factor	*M. incognita*	Tobacco	Decreased number of established nematode	6-7 wpi	Yadav et al., 2006
Secreted peptide 16D10^**^	*M. incognita*	Arabidopsis	Decreased number of galls and gall size	4 wpi	Huang et al., 2006
	*M. javanica*				
	*M. hapla*				
	*M. arneria*				
Major sperm protein, MSP^**^	*H. glycines*	Soybean	Reduced fecundity	8 wpi	Steeves et al., 2006
*MjTIS-11*, Putative transcription factor	*M. javanica*	Tobacco		6 wpi	Fairbairn et al., 2007
*4G06, 3B05, 10A06, 8H07*	*H. schachtii*	Arabidopsis	Decreased number of developing females	14 dpi	Sindhu et al., 2009
Ribosomal protein-3a,4, Spliceosomal SR protein^**^	*H. glycines*	Soybean roots	Reduction in number of females	8 dpi	Klink et al., 2009
*Fib 1, Y 25*	*H. glycines*	Chimeric soybean root system	Suppression of nematode reproduction and development	5 wpi	Li et al., 2010a
*Cpn -1, Y25, Prp-17*	*H. glycines*	Composite soybean plants	Reduction in reproduction and development	5 wpi	Li et al., 2010b
*Mispc3, Miduox*	*M. incognita*	Arabidopsis	Reduction of nematode number in root, retarded female development	20 dpi	Charlton et al., 2010
Tyrosine Phosphatase, Mitochondrial stress -70 protein precursors, Lactate dehydrogenase	*M. incognita*	Soybean roots	Decreased number of galls, 5-6 fold reductions in mature female diameter	28 dpi	Ibrahim et al., 2011
*Mi-Rpn7*	*M. incognita*	Tomato hairy roots	Reduction in reproduction and motility	40 dpi	Niu et al., 2012
Parasitism gene 8D05^**^	*M. incognita*	Arabidopsis	Reduction in gall number	8 wpi	Xue et al., 2013
Calreticulin - *MiCRT*	*M. incognita*	Arabidopsis	Reduction in gall number	8 wpi	Jaouannet et al., 2013
Fatty acid and retinol binding protein (*Mj-far-1*)	*M. javanica*	Tomato	Ceased development of nematodes along with reduction in giant cell number	15 and 28 dpi	Iberkleid et al., 2013
*16D10*	*M. incognita*	Grape hairy roots	Transgenic hairy root lines showed less susceptibility to nematode infection	5 wpi	Yang et al., 2013
FMRFamide-like peptides (*flp-14, flp-18*)	*M. incognita*	Tobacco	Reduction in gall number, fecundity, female development and increased root growth of transgenics	30 dpi	Papolu et al., 2013
Pv010^**^	*Pratylenchus vulnus*	Walnut transformed root	Reduced nematode multiplication with no visible lesions	60 dpi	Walawage et al., 2013
Effector gene, *Mc16D10L*	*M. chitwoodi*	Potato	Reduction in fecundity and pathogenicity	35 and 55 dpi	Dinh et al., 2014a
Effector gene, *Mc16D10L*	*M. chitwoodi*	Arabidopsis, Potato	Reduction in fecundity and pathogenicity	35 and 55 dpi	Dinh et al., 2014b
Effector gene, Gp-hyp^**^	*G. pallida*	Potato hairy root	Reduction in nematode parasitism	2 wpi	Eves-van den Akker et al., 2014

* *wpi, weeks post inoculation; dpi, days post inoculation*.

** *Target mRNA depletion was not detected in the feeding nematodes*.

Based on the outcome of several RNAi studies in PPNs, it seems plausible that host-delivered RNAi can elicit phenotypic changes in the invading PPNs when genes expressed in essential cellular processes of nematodes are targeted. Very few of the parasitism genes targeted show a strong effect on PPN infection, possibly because the majority of effector genes have redundant, combinatorial or additive effects in the plant-nematode interaction. Nevertheless, nematode effectors are a worthwhile group of target genes for *in planta* RNAi strategy as effectors generally lack high homology with the genes of organisms from other taxa (Danchin et al., 2013), thereby diminishing the potential for problems related to off-target effects.

Apart from the migratory endoparasite, *P. vulnus* (Walawage et al., 2013), mostly sedentary endoparasites such as root-knot and cyst nematodes have been targeted for *in planta* RNAi experiments. Among them, *Meloidogyne* spp. dominates the list compared to a few cyst species, including *H. glycines, H. schachtii, and G. pallida*. Other economically important cyst nematodes like *H. avenae* and *G. rostochiensis* have not been targeted yet for *in planta* studies, although the latter was successfully used as a candidate for *in vitro* experiments (Chen et al., 2005). The nematode feeding tube, which functions as a selective barrier to prevent the clogging of the nematode stylet (Hussey and Mims, 1991), may play a significant role in the uptake of dsRNA molecules during *in vivo* feeding of sedentary endoparasites on transgenic plants. As the size exclusion limit of the feeding tube is considerably higher in *Meloidogyne* spp. than that of cyst nematodes (Bockenhoff and Grundler, 1994; Urwin et al., 1997; Lilley et al., 2007; Dinh et al., 2014b), root-knot nematodes are presumably better candidates for application of host-delivered RNAi.

Although several studies have reported the detection of target gene siRNAs in plant roots through northern analysis (Huang et al., 2006; Steeves et al., 2006; Fairbairn et al., 2007; Sindhu et al., 2009; Li et al., 2010b; Papolu et al., 2013; Yang et al., 2013; Dinh et al., 2014a), that fact that the same could not be accomplished in a few instances may be due to the limited sensitivity of northern blot assays (Charlton et al., 2010; Dinh et al., 2014b). In either case however, the pathway through which the RNAi effect occurs could not be established. Others have employed qRT-PCR to study the expression of intron/spacer region of the hairpin construct for the detection of unprocessed transcript (Patel et al., 2008, 2010). Yang et al. (2013) have speculated that hairpin constructs with shorter loops are more abundantly transcribed than longer ones as longer stems may hinder dsRNA processing by Dicer enzyme, thus reducing siRNA production. Therefore, precision in designing loop length and sequence of the construct is necessary to maximize RNAi silencing efficacy.

In spite of several drawbacks, RNAi can be far more effective in plant-nematode interface. In plants and in *C. elegans*, primary siRNAs generated by the cleavage of exogenous dsRNA can lead to the synthesis of secondary siRNAs from the target mRNA, mediated by the RNA-dependent RNA polymerase (RdRP) enzyme. With this additional step of amplification, secondary siRNAs become more abundant than the primary siRNAs and show higher efficacy in post-transcriptional gene silencing (Rosso et al., 2009). RNAi can also be advantageous if the target gene and its corresponding dsRNA construct are co-expressed in the same transgenic plant. Crossing of an Arabidopsis line expressing *16D10* gene of *M. incognita* with another line expressing RNAi construct of *16D10*, exhibited a higher level of *16D10* siRNA accumulation in the progeny plants, thereby leading to superior nematode resistance (Huang et al., 2006). Crossing of two Arabidopsis lines expressing dsRNA of *Miduox* and *Mispc3* gene led to an additive effect that further reduced *M. incognita* development in the progeny plants (Charlton et al., 2010). Gene stacking technology was also used to generate RNAi-mediated combined resistance against *P. vulnus* infection and crown gall disease in walnut (Walawage et al., 2013). Therefore, simultaneous interference of multiple genes enhances the efficacy and durability of RNAi in PPNs that feed on the plants expressing multiple dsRNA constructs.

Breeding for nematode resistance is a time-consuming strategy that involves additional complications like pre- and post-zygotic incompatibility barriers and biosafety-related issues associated with transgenics expressing nematicidal proteins have not yet been resolved. As no foreign proteins are expressed *in planta* in host-delivered RNAi (Atkinson et al., 2012) RNAi-based strategies offer an attractive alternative. Besides Arabidopsis, agricultural crops (tobacco, soybean, tomato, potato, grape, and walnut) have also been engineered for RNAi against PPNs, although economically-important crops such as cereals and pulses have yet to be tested.

## Conclusion and future prospects

RNAi has emerged as a potent and successful technology for crop protection in recent years but there remain certain limitations that need to be addressed before adopting this technology in the field. One major concern regarding the employment of RNAi-based nematode management strategy is the potential for off-target effects. As the RNAi mechanism occurs in a highly sequence-specific manner, cross-hybridization with endogenous transcripts having partial homology to the introduced dsRNA molecule may lead to silencing of non-target genes, which may have effects in non-targeted organisms. Some improvements to avoid off-target effects include, (i) in depth *in silico* homology searches to identify off-target sequences and eliminating such sequences in RNAi constructs, (ii) root-specific or nematode-inducible promoters should be used so that siRNAs are not expressed in the edible parts of the plant, (iii) avoid targeting gene families that exhibit high degrees of sequence conservation across plant and animal kingdoms, (iv) sequences from 5′ or 3′ untranslated regions (UTR) should be used as siRNA targets, as they are usually less conserved than coding regions, (v) species-specific targets should be screened through a comparative genomics approach utilizing data generated from genomics or transcriptomics of PPNs and their hosts, and (vi) most importantly, RNAi constructs should target several nematode genes.

Although significant progress has been made in engineering plant resistance against harmful nematodes through RNAi approaches, most of the studies did not report complete resistance against PPNs. One possible way to improve this is by using chimeric RNAi constructs targeting multiple genes involved in multiple cellular functions. Likewise, the use of plant promoters that are tissue specific, wound-inducible or PPN-inducible may also achieve improved resistance. Once this novel resistance mechanism is perfected, this technology will create a new era in PPN management and its application could be extended to commercial crops.

### Conflict of interest statement

The authors declare that the research was conducted in the absence of any commercial or financial relationships that could be construed as a potential conflict of interest.
